# Pre-diagnosis Cruciferous Vegetables and Isothiocyanates Intake and Ovarian Cancer Survival: A Prospective Cohort Study

**DOI:** 10.3389/fnut.2021.778031

**Published:** 2021-11-24

**Authors:** Yi-Fan Wei, Ying-Ying Hao, Song Gao, Xiu-Qin Li, Fang-Hua Liu, Zhao-Yan Wen, Han-Yuan Wang, Shuang Zhang, Shi Yan, Meng Luan, Yu-Hong Zhao, Ting-Ting Gong, Qi-Jun Wu

**Affiliations:** ^1^Department of Clinical Epidemiology, Shengjing Hospital of China Medical University, Shenyang, China; ^2^Clinical Research Center, Shengjing Hospital of China Medical University, Shenyang, China; ^3^Department of Obstetrics and Gynecology, Shengjing Hospital of China Medical University, Shenyang, China

**Keywords:** cohort, cruciferous vegetables, isothiocyanates, ovarian cancer, prognosis, survival

## Abstract

**Background:** The associations of the consumption of cruciferous vegetables (CVs) and their bioactive components, isothiocyanates (ITCs), with ovarian cancer (OC) mortality have been unclear, owing to limited studies and inconsistent findings. To date, no studies have evaluated these associations among Chinese patients with OC. This study aims to provide more evidence indicating the relationships of pre-diagnosis CVs and ITC intake with OC survival.

**Methods:** We examined the associations of pre-diagnosis CV and ITC intake with OC mortality in a hospital-based cohort (*n* = 853) of Chinese patients with epithelial OC between 2015 and 2020. Pre-diagnosis dietary information was evaluated with a validated food frequency questionnaire. Deaths were ascertained until March 31, 2021 *via* medical records and active follow-up. The associations were examined with the Cox proportional hazards model, adjusted for potential confounders, and stratified by menopausal status, residual lesions, histological type, and body mass index (BMI).

**Results:** During a median follow-up of 37.2 months (interquartile: 24.7–50.2 months), we observed 130 deaths. The highest tertile of total CV intake was associated with better survival than the lowest tertile intake [hazard ratio (HR) = 0.57, 95% confidence interval (CI) = 0.33–0.98, *p* trend < 0.05]. In addition, higher intake of ITCs from CVs was associated with better survival (HR_T3VS.T1_ = 0.59, 95% CI = 0.36–0.99, *p* trend = 0.06). Significant inverse associations were also observed for subgroup analyses stratified by menopausal status, residual lesions, histological type, and BMI, although not all associations showed statistical significance.

**Conclusion:** Increasing pre-diagnosis consumption of CVs and ITCs was strongly associated with better survival in patients with OC.

## Introduction

Ovarian cancer (OC) is a gynecological malignant tumor with insidious onset, rapid development, and poor prognosis (1). Worldwide, 313,959 women were diagnosed with OC, and 207,252 new deaths due to this disease occurred in 2020 (2). In China, there were ~25,000 new cases and 22,000 new deaths in 2015 (3). Fewer than half of affected women survive beyond 5 years after diagnosis because of the predominance of aggressive high-grade serous carcinomas and the absence of specific early symptoms and effective early detection strategies (1). Emerging evidence suggests that maximal cytoreductive surgery (4), statin use (5), obesity (6), and vegetable consumption (7) might affect OC survival.

Previous research has suggested that of vegetables, particularly cruciferous vegetables (CVs), might increase OC survival (8). CVs come from plants in the Cruciferae and Brassicaceae families, which account for a large proportion of vegetables, e.g., broccoli, brussels sprouts, cabbage, cauliflower, collard greens, kale, kohlrabi, mustard, rutabaga, turnips, bok choy, and Chinese cabbage (9). Isothiocyanates (ITCs) are formed from the hydrolysis of glucosinolates in plants through the action of the enzyme myrosinase and the gut microbiota, and they are abundant in CVs (10, 11). Different glucosinolates in CVs produce distinct ITCs (11). Some ITCs are volatile and become hydrolyzed at higher cooking temperatures (12, 13). Previous epidemiological studies have indicated that CVs are inversely associated with several health outcomes including cardiovascular disease (14), type 2 diabetes mellitus (15), gastrointestinal cancer (16, 17), breast cancer (18), and reproductive cancers (19, 20). Several epidemiological studies have also shown that ITCs might be associated with various cancers (21–23), diabetes (24, 25), and cardiovascular disease (26, 27). Notably, only three observational studies have focused on the association between pre-diagnosis CV intake and OC mortality; however, these findings were inconsistent. For example, two studies found that a diet high in CVs might help improve OC survival (8, 28). However, one study on 811 patients with OC found no association between CV intake and OC survival (29). More importantly, no study has investigated the relationship between pre-diagnosis ITC intake and OC prognosis.

To provide more evidence indicating the relationships of pre-diagnosis CVs and ITC intake with OC survival, we conducted a hospital-based OC follow-up study in China. To our knowledge, this study is the first large-scale prospective study exploring this topic among Chinese women with different dietary habits, as compared with Europeans and Americans.

## Materials and Methods

### Study Population

**Figure 1:**
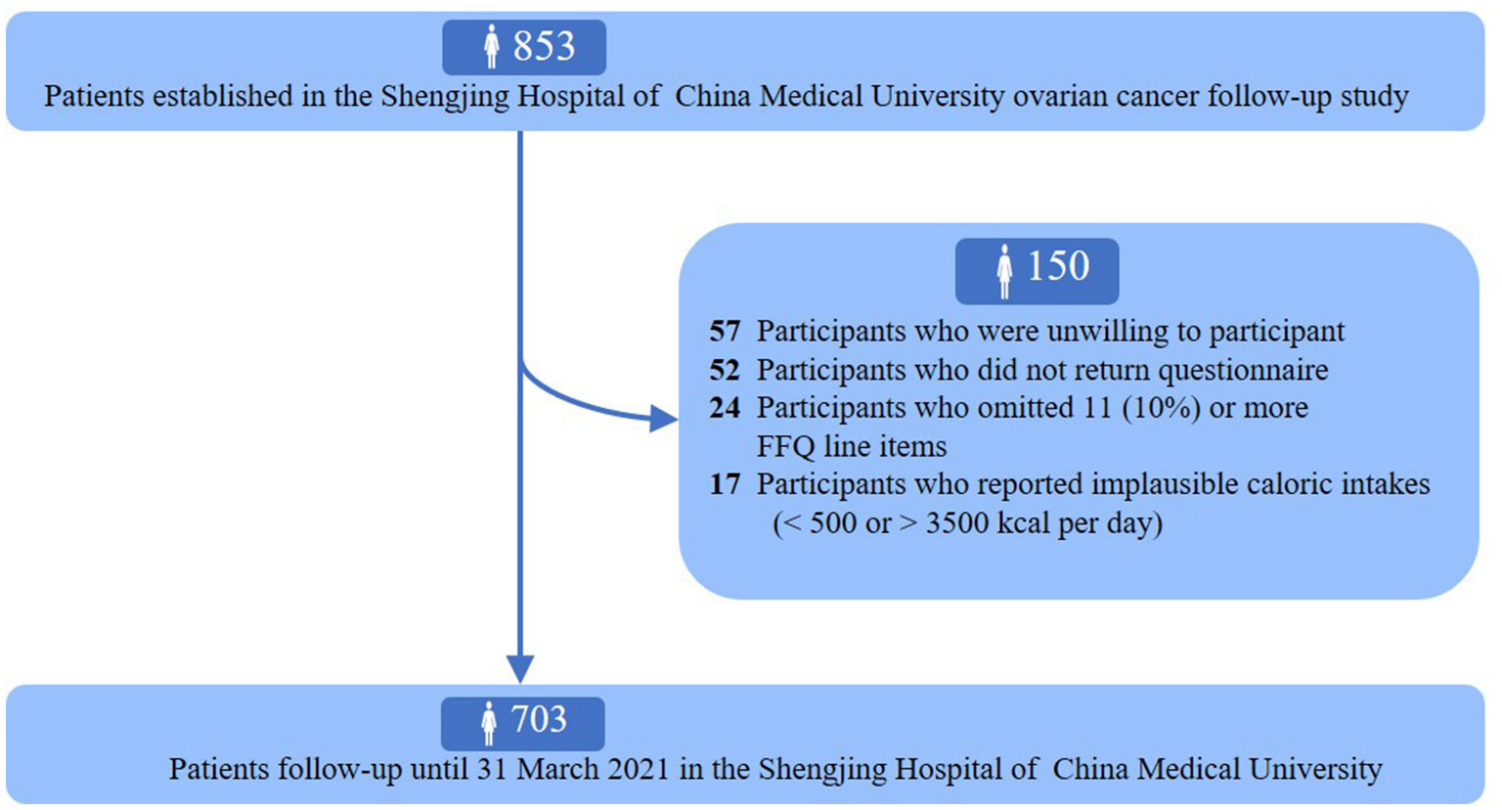
Flow of participants through study.

The ovarian cancer follow-up study (OOPS) is a prospective longitudinal cohort study of patients newly diagnosed with OC. This study aimed to collect demographic, clinical, and lifestyle data from patients with OC to assess their associations with cancer-related outcomes, and was approved by the Institutional Review Board of the Ethics Committee of Shengjing Hospital of China Medical University, Shenyang, China. Information collection on long-term outcomes remains ongoing. Between January 2015 and December 2020, 853 patients with OC who were 18–75 years of age were enrolled. Among them, 796 women agreed to participate, and 744 (93%) returned the completed study questionnaire. We further excluded participants who left 11 (10%) or more food items blank (*n* = 24) and those with implausible caloric intakes (<500 or >3,500 calories per day) (*n* = 17). Finally, dietary data were available for 703 women with OC (Figure 1).

### Data Collection

Through self-administered questionnaires, we collected data on demographics and lifestyle factors, including educational level, monthly household income, physical activity (work, commuting, household chores, and leisure-time exercise) (30), smoking status, alcohol drinking, and tea drinking. Height and body weight [used to calculate body mass index (BMI)] were measured by trained staff following a standard protocol. In addition, clinical variables were abstracted from the electronic medical records of the Shengjing hospital information system. These variables included age at diagnosis, histological type (serous or non-serous), histopathologic grade (well, moderate, and poorly differentiated), International Federation of Gynecology and Obstetrics (FIGO) stage (I–II, III–IV, and unknown), residual lesions (none, <1, and ≥1 cm), and comorbidities (hypertension, coronary heart disease, diabetes, and so on) (31, 32).

### Dietary Exposure Assessment

Dietary intake was assessed at recruitment with a 111-item food frequency questionnaire (FFQ), which has been verified to have reasonable reliability and validity. The reproducibility coefficients (spearman correlation coefficients and intraclass correlation coefficients) were above 0.5 for most food groups, and correlation coefficients (spearman correlation coefficients) were between 0.3 and 0.7 for most food groups between the FFQ and weighed diet records. Participants reported their usual frequency of intake for standard serving sizes of food items in the 12 months before OC diagnosis. Frequency categories included almost never, two or three times per month, one time per week, two or three times per week, four to six times per week, one or two times per day, and more than two times per day. In our study, CV intake was calculated by summing the intake amounts of Chinese cabbage, pakchoi, kohlrabi, rape, broccoli, cauliflower, and raphanus sativus. Intake of each CV in grams/day was calculated through multiplying consumption frequencies per day by fitted portion sizes (g/time). Spearman correlation coefficients and intraclass correlation coefficients of reproducibility for CV were 0.61 and 0.53, respectively. The crude and energy-adjusted spearman correlation coefficients of validity for CV were 0.43 and 0.33, respectively. The Chinese Food Composition Tables (33) were used as the nutrient database to calculate the daily ITC intake. ITC intake was calculated by first multiplying the amount (in grams) consumed (for each CV) by its nutrient content per gram and then adding the ITC contributions across all CVs (34–36).

### Follow-Up and Outcomes

Information on the vital status of OOPS participants was ascertained from medical records and active follow-up. The outcome of focus was death due to any cause. Survival time was defined as the interval between histologic diagnosis and the date of death due to any cause or the date of the last follow-up (March 31, 2021) for patients who were still alive.

### Statistical Analysis

Differences in general and clinical characteristics according to CV intake categories were assessed with the χ^2^ test, except for continuous variables, which were assessed with the one-way ANOVA or the Kruskal–Wallis test. The Kaplan–Meier technique was used to plot crude survival curves and estimate the crude overall survival probabilities. Cox proportional hazards regression models were used to assess the associations of pre-diagnosis CVs and ITC intake with overall survival. The proportional hazards assumption was tested through inclusion of an interaction term between each activity variable and log survival time, and no violations were found (all *p* > 0.05). CV and ITC intake was categorized by tertile distribution, wherein the lowest tertile served as the reference group. Furthermore, we tested the association between the frequency of specific species CVs (including Chinese cabbage, pakchoi, kohlrabi, rape, broccoli, cauliflower, and raphanus sativus) and OC survival. The linear trend tests were performed by assigning the median value of consumption for each tertile of CVs and ITCs and treating it as a continuous variable in the respective regression model (37). In model 1, we did not adjust any covariates to calculate crude hazard ratio (HR) and 95% confidence interval (CI). In order to account for the influence of age at diagnosis and total energy, we adjusted for age at diagnosis (<50 or ≥50 years) and total energy intake (continuous, kcal) in model 2. In addition, we further adjusted for BMI (continuous, kg/m^2^), comorbidities (yes or no), dietary changes (yes or no), meat (continuous, g/days), fruit (continuous, g/days), green leafy vegetables (continuous, g/days), alliums (continuous, g/days), soy (continuous, g/days), education (junior secondary or below, senior high school/technical secondary school, and junior college/university or above), FIGO stage (I–II, III–IV, and unknown), histological type (serous or non-serous), histopathologic grade (well, moderate, and poorly differentiated), menopausal status (yes or no), parity (≤ 1, ≥2), physical activity (continuous), residual lesions (none, <1, and ≥1 cm), and smoking status (yes or no) in model 3 to minimize the impact of clinical characteristics, lifestyle factors, and dietary factors on the survival. Of these covariates, comorbidities were defined as the simultaneous presence of two or more diseases in some individuals (32). In our analysis, the comorbidities include hypertension, coronary heart disease, diabetes, and so on. Dietary change was collected *via* self-administered questionnaires, and participants were asked whether they have changed their dietary habits recently. Parity was also gathered through self-administered questionnaires. Based on previous study, we assessed physical activity in these OC patients (38). Briefly, participants were asked the usual type and duration of activities related to work, commuting, household chores, and leisure-time exercise during the past 12 months in our study (38). We further used metabolic equivalent tasks (METs) from the 2011 update (30) of a major compendium of physical activities to calculate the amount of physical activity.

Stratified analyses were used to assess potential effect modification by menopausal status (no compared with yes), residual lesions (no compared with yes), histological type (serous compared with non-serous), and BMI (<25 compared with ≥25 kg/m^2^). Potential interactions of CV and ITC intake with these stratifying variables were assessed by the addition of cross-product terms to the multivariable Cox models. We also analyzed the relationships of pre-diagnosis CVs and ITCs with overall survival in stage III or IV patients with OC. All analyses were performed in SAS version 9.4 (SAS Institute, Cary, NC, USA). Two-sided *P* < 0.05 were considered statistically significant.

## Results

The distribution of socio-demographic and clinical characteristics of 703 patients with OC according to tertiles of total CVs is presented in Table 1. The median (interquartile) age at diagnosis was 53.0 (48.0–60.0) years, and no significant difference was observed among the three groups. During a median follow-up of 37.2 months (interquartile: 24.7–50.2 months), we observed 130 deaths, and patients with a higher CV intake had longer follow-up times (*p* < 0.05). In addition, patients with a higher intake of CVs tended to consume more meat, green leafy vegetables, allium vegetables, fruits, beans and bean products, tea, and total energy. Furthermore, the proportion of postmenopausal women was significantly higher among patients with greater CV intake. Table 2 shows that patients with a non-serous histological subtype, later FIGO stage, and larger residual lesions had poorer survival in this study.

**Table 1:** General characteristics of ovarian cancer patients according to cruciferous vegetables (*N* = 703).

**Variables**	**Total cruciferous vegetables (g/day)**			***P*** **-value**
	**T1 (<33.08)**	**T2 (33.08–70.20)**	**T3 (≥70.20)**	
**No. of patients**	234	234	235	
**Age at diagnosis (years), Median (IQR)**	53.00 (47.00–60.00)	53.00 (48.00–59.00)	53.00 (48.00–61.00)	0.37
**Follow-up time (m), Median (IQR)**	29.37 (18.10–44.10)	30.59 (21.67–44.03)	34.27 (22.50–49.70)	<0.05
**Body mass index (kg/m^2^), Median (IQR)**	23.30 (20.70–25.10)	23.30 (21.20–24.90)	22.90 (20.60–25.10)	0.67
**Physical activity (MET/hours/days), Median (IQR)**	13.55 (7.00–21.40)	15.10 (7.30–22.90)	12.90 (5.70–22.30)	0.35
**Diet intake (Mean ± SD)**
Total energy (kcal/d)	1,210.39 ± 446.59	1,399.13 ± 448.19	1,756.46 ± 602.84	<0.05
Meat (g/day)	32.36 ± 27.49	34.90 ± 26.28	41.85 ± 33.41	<0.05
Green leafy vegetables (g/day)	4.17 ± 4.40	8.57 ± 9.12	15.35 ± 10.66	<0.05
Allium vegetables (g/day)	20.50 ± 13.64	26.94 ± 16.47	42.11 ± 27.55	<0.05
Fruits (g/day) (g/day)	132.20 ± 104.22	180.88 ± 141.85	270.50 ± 184.37	<0.05
Soy (g/day)	54.30 ± 55.27	77.89 ± 60.34	123.45 ± 96.36	<0.05
**Diet change (** ***n*** **, %)**				0.32
No	185(79.06)	171 (73.08)	179 (76.17)	
Yes	49 (20.94)	63(26.92)	56 (23.83)	
**Smoke status (** ***n*** **, %)**				0.43
No	208 (88.89)	216 (92.31)	211 (89.79)	
Yes	26 (11.11)	18 (7.69)	24 (10.21)	
**Alcohol intake (** ***n*** **, %)**				0.52
No	179 (76.50)	189 (80.77)	186 (79.15)	
Yes	55 (23.50)	45 (19.23)	49 (20.85)	
**Tea drinking (** ***n*** **, %)**				<0.05
No	168 (71.79)	165 (70.51)	144 (61.28)	
Yes	66 (28.21)	69 (29.49)	91 (38.72)	
**Menopausal status (** ***n*** **, %)**				<0.05
No	79 (33.76)	63 (26.92)	53 (22.55)	
Yes	155 (66.24)	171 (73.08)	182 (77.45)	
**Parity (** ***n*** **, %)**				0.54
≤ 1	173 (73.93)	169 (72.22)	163 (69.36)	
≥ 2	61 (26.07)	65 (27.78)	72 (30.64)	
**Educational level (** ***n*** **, %)**				0.16
Junior secondary or below	124 (52.99)	114 (48.72)	137 (58.30)	
Senior high school/technical secondary school	47 (20.09)	60 (25.64)	40 (17.02)	
Junior college/university or above	63 (26.92)	60 (25.64)	58 (24.68)	
**Income per month (Yuan), (** ***n*** **, %)**				0.61
<5,000	139 (59.40)	139 (59.40)	143 (60.85)	
5,000 to <10,000	64 (27.35)	71 (30.34)	59 (25.11)	
≥10,000	31 (13.25)	24 (10.26)	33 (14.04)	
**Age at diagnosis (Year), (** ***n*** **, %)**				0.87
≤ 50	89 (38.03)	85 (36.32)	84 (35.74)	
>50	145 (61.97)	149 (63.68)	151 (64.26)	
**Histological type (** ***n*** **, %)**				0.74
Serous	155 (66.24)	161 (68.80)	163 (69.36)	
Non-serous	79 (33.76)	73 (31.20)	72 (30.64)	
**Histopathologic grade (** ***n*** **, %)**				0.73
Well differentiated	21 (8.97)	19 (8.12)	16 (6.81)	
Moderately differentiated	19 (8.12)	13 (5.56)	16 (6.81)	
Poorly differentiated	194 (82.91)	202 (86.32)	203 (86.38)	
**FIGO stage (** ***n*** **, %)**				0.28
I–II	121 (51.71)	100 (42.74)	121 (51.49)	
III–IV	105 (44.87)	126 (53.84)	107 (45.53)	
Unknown	8 (3.42)	8 (3.42)	7 (2.98)	
**Residual lesions (** ***n*** **, %)**				0.75
No	181 (77.35)	189 (80.77)	183 (77.87)	
<1 cm	40 (17.09)	30 (12.82)	36 (15.32)	
≥1 cm	13 (5.56)	15 (6.41)	16 (6.81)	
**Comorbidities (** ***n*** **, %)**				0.37
No	139 (59.40)	124 (52.99)	130 (55.32)	
Yes	95 (40.60)	110 (47.01)	105 (44.68)	

*IQR, interquartile range; MET, metabolic equivalent task; SD, standard deviation; T, tertile*.

*P-values were determined with one-way ANOVA or the Kruskal–Wallis test for continuous variables, and the chi-square test for categorical variables*.

**Table 2:** Selected clinical characteristics and associations with all-cause mortality among women diagnosed with ovarian cancer (*N* = 703).

**Characteristic**	**No. of deaths/total (%)**	**Crude HR (95% CI)**	**Adjusted HR^a^ (95% CI)**
**Age at diagnosis**			
≤ 50	45/258 (17.44)	1.00 (ref)	1.00 (ref)
>50	85/445 (19.10)	1.18 (0.82–1.70)	1.24 (0.85–1.79)
**Histological type**			
Serous	92/479 (19.21)	1.00 (ref)	1.00 (ref)
Non-serous	38/224 (16.96)	0.87 (0.59–1.27)	1.71 (1.11–2.66)
**Histopathologic grade**			
Well differentiated	5/56 (8.93)	1.00 (ref)	1.00 (ref)
Moderately differentiated	7/48 (14.58)	1.44 (0.46–4.57)	1.12 (0.35–3.57)
Poorly differentiated	118/599 (19.70)	2.32 (0.95–5.67)	1.76 (0.70–4.43)
**FIGO stage**			
I–II	41/342 (11.99)	1.00 (ref)	1.00 (ref)
III–IV	89/338 (26.33)	2.75 (1.89–4.00)	2.54 (1.65–3.91)
**Residual lesions**			
No	82/553 (14.83)	1.00 (ref)	1.00 (ref)
<1 cm	31/106 (29.25)	2.22 (1.47–3.36)	1.73 (1.11–2.68)
≥1 cm	17/44 (38.64)	3.18 (1.89–5.37)	2.41 (1.39–4.16)
**Comorbidities**			
No	74/393 (18.83)	1.00 (ref)	1.00 (ref)
Yes	56/310 (18.06)	0.82 (0.58–1.16)	0.97 (0.68–1.38)

a *Mutually adjusted for all other variables listed in the table*.

*CI, confidence interval; HR, hazard ratio; ref, reference*.

**Figure 2:**
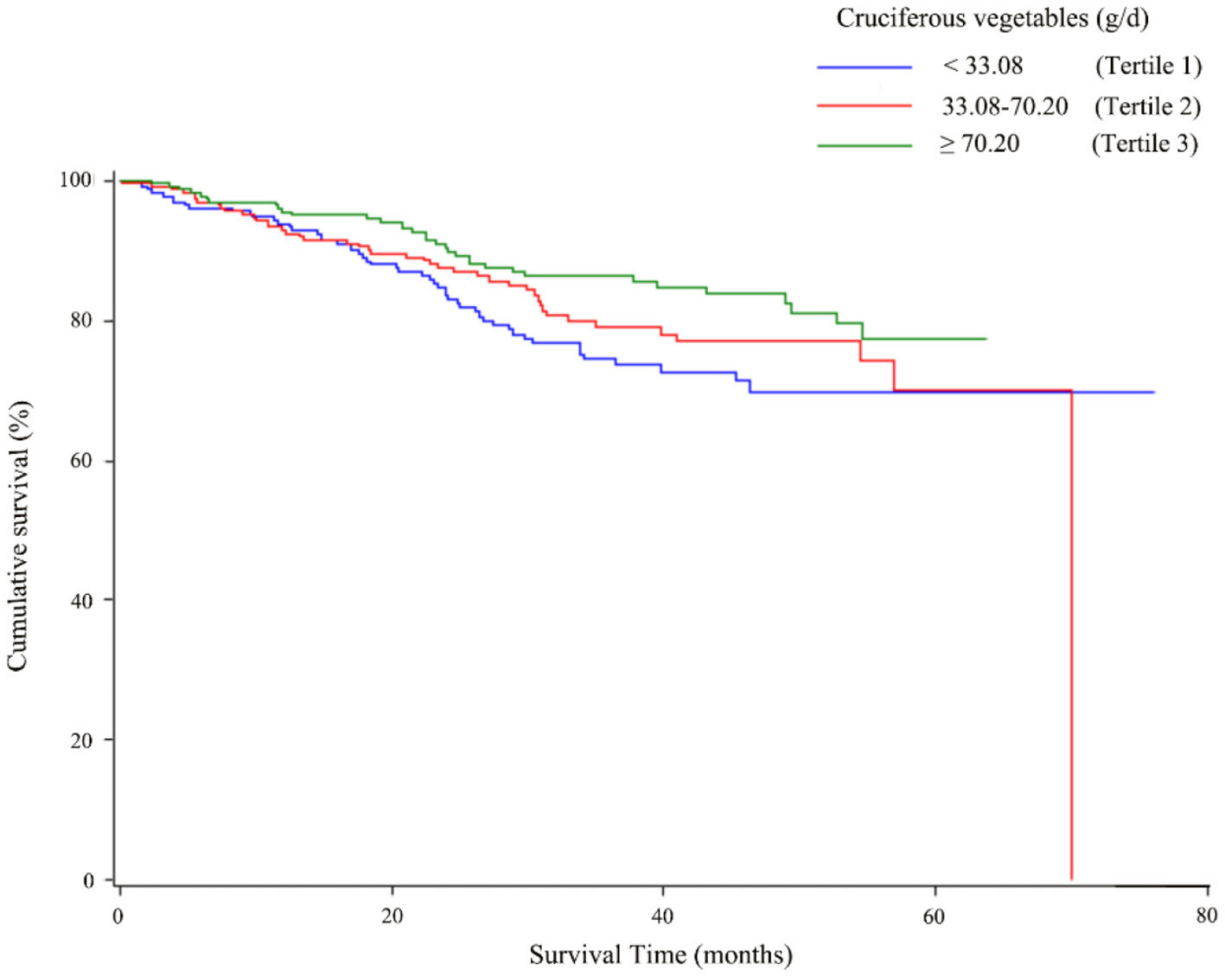
Kaplan–Meier survival curves for total cruciferous vegetables intake.

The associations of total CV as well as ITC intake with the overall survival of OC are described in Table 3. The highest tertile of total CV intake was associated with better survival than the lowest tertile intake (HR = 0.57, 95% CI = 0.33–0.98), and a linear trend was also evident (*p* trend < 0.05). As shown in Figure 2, the OC survival was better in patients with the highest tertile of CV intake than those in the lowest tertile. Moreover, a similar protective effect was observed for ITCs from CV intake (HR _T3VS.T1_ = 0.59, 95% CI = 0.36–0.99), although we did not observe a significant linear trend (*p* trend = 0.06). Analyses of specific species of CVs indicated that the significant protective effect of Chinese cabbage, kohlrabi, and pakchoi was consistent with the main findings (Supplementary Table 1).

**Table 3:** Hazard ratio (95% CI) for overall survival among ovarian cancer patients according to cruciferous vegetables and relative nutrient intake.

**Dietary variables**	**T1**	**T2**	**T3**	***P*** ** for trend^d^**
**Total cruciferous vegetables (g/day)**				
Rang of intake	<33.08	33.08–70.20	≥70.20	
Deaths, *N* (% of total deaths)	52 (40)	44 (33.85)	34 (26.15)	
Model 1^a^ HR (95% CI)	1.00 (ref)	0.82 (0.55–1.22)	0.59 (0.38–0.91)	<0.05
Model 2^b^ HR (95% CI)	1.00 (ref)	0.77 (0.51–1.16)	0.50 (0.31–0.81)	<0.05
Model 3^c^ HR (95% CI)	1.00 (ref)	0.81 (0.53–1.24)	0.57 (0.33–0.98)	<0.05
**Isothiocyanates (μmol/day)**				
Rang of intake	<3.90	3.90-9.44	≥9.44	
Deaths, *N* (% of total deaths)	57 (43.85)	36 (27.69)	37 (28.46)	
Model 1^a^ HR (95% CI)	1.00 (ref)	0.59 (0.39–0.90)	0.57 (0.38–0.86)	<0.05
Model 2^b^ HR (95% CI)	1.00 (ref)	0.56 (0.37–0.86)	0.49 (0.30–0.77)	<0.05
Model 3^c^ HR (95% CI)	1.00 (ref)	0.66 (0.43–1.02)	0.59 (0.36–0.99)	0.06

*CI, confidence interval; HR, hazard ratio; ref, reference; T, tertile*.

a *Model 1 unadjusted*.

b *Model 2 adjusted for age at diagnosis and total energy*.

c *Model 3 same as Model 2 and further adjusted for body mass index, comorbidities, diet change, education, FIGO stage, histological type, histopathologic grade, menopausal status, parity, physical activity, residual lesions, smoke status, meat, fruit, green leafy vegetables, and allium vegetables*.

d *P-value for linear trend calculated from category median values*.

**Figure 3:**
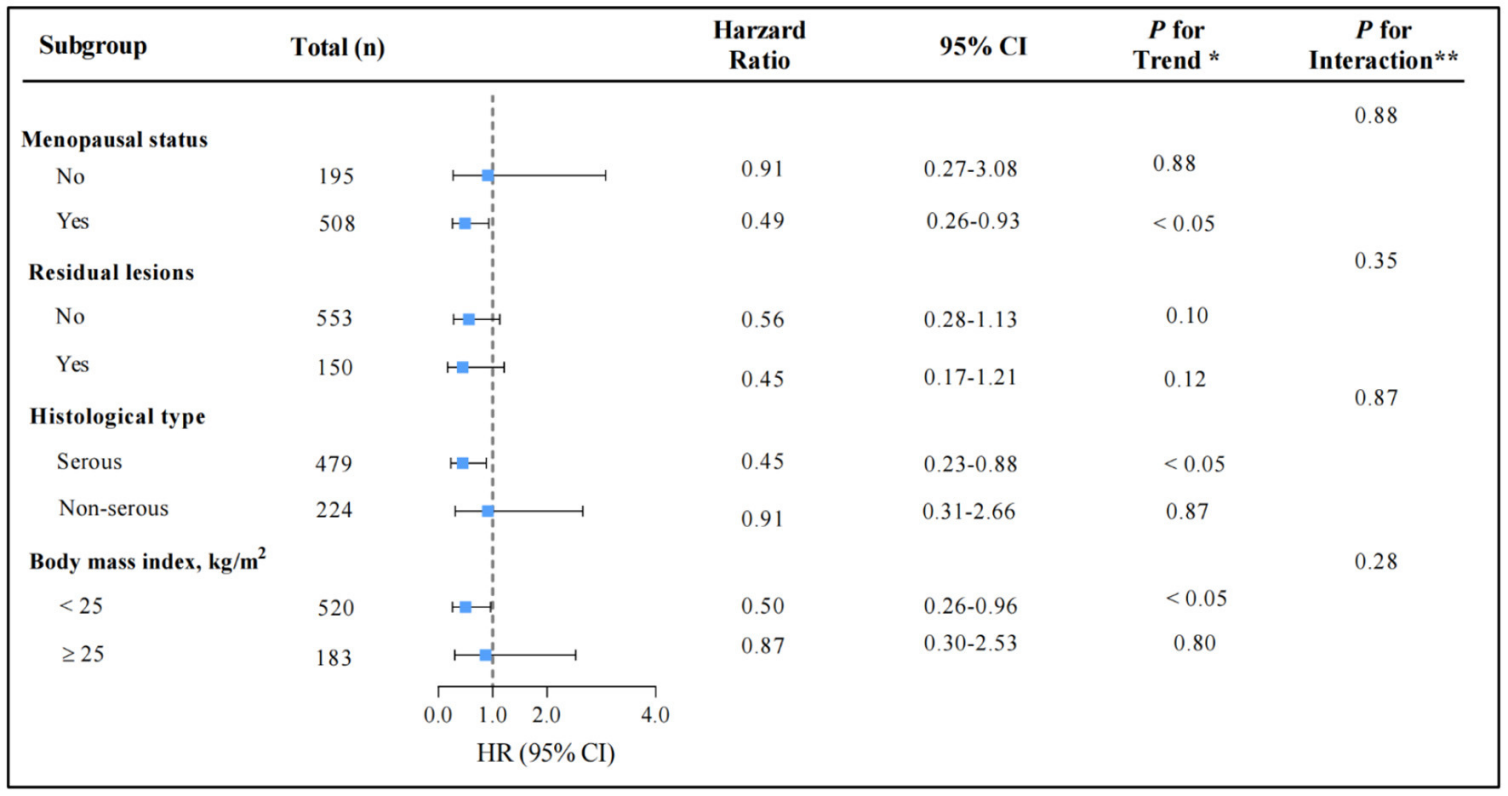
Multivariable hazard ratios (HRs) and 95% CIs for overall survival among ovarian cancer patients across strata of various factors. The analyses used three categories of cruciferous vegetables intake (T_1_ < 33.08, T_2_ 14.76–90.00, and T_3_ ≥ 90.00 g/d). The forest plot represents the HRs of the comparison of the highest vs. the lowest of cruciferous vegetables intake. Cox model stratified by menopausal status, residual lesions, histological type, and body mass index, with additional adjustments for age at diagnosis, body mass index, comorbidities, diet change, education, FIGO stage, histological type, histopathologic grade, menopausal status, parity, physical activity, residual lesions, smoke status, meat, fruit, green leafy vegetables, allium vegetables, and total energy. *indicates *P* for trend across levels of cruciferous vegetables intake. **indicates *P* for interaction between strata and cruciferous vegetables intake. *P*-values are two-sided.

The relationship between total CV intake and overall survival in patients with OC was evaluated across potential effect-modifying variables, such as menopausal status, residual lesions, histological subtype, and BMI. The better survival advantages associated with the highest total CV intake were present only in postmenopausal patients, patients with serous histological subtype, or patients with BMI <25 (Figure 3). Additionally, we examined the influence of ITC intake on overall OC survival across the aforementioned potential effect-modifying variables that generated similar patterns (Supplementary Figure 1).

## Discussion

The present findings from this prospective cohort study of 853 Chinese women diagnosed with OC supported that pre-diagnosis CV and ITC intake might enhance the survival of patients with OC. Additionally, we observed significant findings in the analysis of specific species of CVs including Chinese cabbage, kohlrabi, and pakchoi.

Evidence from previous studies evaluating pre-diagnosis CV intake in relation to mortality among OC survivors has been limited and inconsistent. Our findings were consistent with some previous results. For example, Dolecek et al. (28) have reported that higher pre-diagnosis CV intake was significantly associated with longer survival in 341 patients with OC (HR = 0.56, 95% CI = 0.37–0.84). In addition, Nagle et al. (8) have presented findings from 609 women with invasive epithelial OC in Australia, in which pre-diagnosis CV consumption was inversely associated with total mortality (HR = 0.75, 95% CI = 0.57–0.98). In contrast, Playdon et al. (29) have reported a null finding (high to low intake, HR = 0.99, 95% CI = 0.77–1.29) in the results of the Australian OC study (*n* = 811), which recruited 18–79 year old patients with OC between January 2002 and June 2006. However, interestingly, they observed a trend toward lower mortality risk with higher CV intake when the analysis was restricted to high-grade serous OC cases. These inconsistent findings might be attributable to several reasons, including different demographic and clinical characteristics of patients with OC, exposure assessment, dietary habits, and potential confounders adjustment. For example, Playdon et al. included patients with more advanced OC (III–IV: 71.0%) than our study (III–IV: 48.1%). The diagnostic age composition of patients in our study ( ≤ 50 years: 36.7%) differed from that in the study of Playdon et al. (<50 years: 18.0%). Additionally, although both these studies used an FFQ to collect the diet information, the specific species of CVs differed among them. Of note, the consumption of CVs also differed among these studies. For example, the lower limit of the highest category in the studies of Dolecek et al., Nagle et al., and Playdon et al., was three servings/week (34.28 g/day), 0.83 servings/day (66.40 g/day), and 1.5 servings/day (120.00 g/day), respectively (1 serving equals to 80 g) (39). In contrast, the lower limit of the highest category for CV intake was 70.20 g/day in the present study. Notably, previous evidence has suggested that consumption of CVs differs among Chinese (>100 g/d), Australian (40–80 g/d), and American (25–30 g/d) populations (40). Furthermore, the difference in the potential confounding adjustments among these studies should not be ignored.

The inverse associations of CV and ITC intake with OC mortality are biologically plausible. CVs are good sources of glucosinolates, which can be hydrolyzed by the plant enzyme myrosinase into biologically active compounds such as ITCs. Findings from previous *in vivo* or *in vitro* studies have shown that ITCs have cancer-preventive potential (41) through the key mechanisms of inducing carcinogen detoxification and elimination through elevated NRF2 signaling (42, 43), enhancing DNA damage repair (44, 45), and eliminating cancer stem cells (46). Different types of ITCs can also achieve anti-OC ability by inhibiting cell proliferation (47–49) and enhancing cisplatin sensitivity (50, 51). Additionally, CVs contain antioxidants, such as carotenoids, vitamin C, and vitamin E. Antioxidants can counteract the effects of reactive oxygen species, including initiation lipid peroxidation, cell damage, disruption of cell signaling, and increased viral replication and expression, all of which are pivotal events in carcinogenesis (52, 53).

This study has several crucial strengths. First, this is the first investigation to report a survival advantage among patients with OC with higher intake of pre-diagnosis CVs and related ITCs in China. Second, the OOPS study was a well-designed prospective cohort study with high baseline survey participation rates and follow-up retention rates, thus minimizing the potential for recall bias or selection bias. Further, we used a validated FFQ to evaluate dietary information through face-to-face interviews, thus providing stable estimates of CV intake. Third, a wide range of potential confounders associated with OC prognosis were strictly adjusted for, such as residual lesions, histological type, and FIGO stage. Fourth, Asian populations habitually consume large amounts of CVs and other plant-based foods, thus enabling hypotheses associated with the underlying health properties of these foods to be investigated specifically (54).

However, potential study limitations also must be considered. First, FFQ was used to evaluate CV intake of OC patients. The standard size of food share was applied to calculate food intake, but the intake of patients could not be accurately assessed. Thus, information bias is inevitable. However, we used a highly reproducible validated FFQ and selected highly trained and skilled researchers to collect dietary information. Additionally, spearman correlation coefficients and intraclass correlation coefficients of reproducibility for CV were 0.61 and 0.53, respectively. Second, compared with the average intake of CVs (~22.6 g/d) in Western populations (55), the intake in Chinese populations exceeds 100 g/d (40), thus potentially limiting the generalizability of our findings to the consumption of CVs in Western populations. Furthermore, ITC exposure levels are influenced by various cooking methods (9). Boiling CVs leads to a 30–60% loss of complete glutathione S-transferases, owing to leaching and thermal degradation (56). Nevertheless, none of the studies have distinguished CV intake according to food preparation method. Hence, further studies must focus on whether the inverse correlation of CV intake might be influenced by the cooking method. Third, the relationship between CV intake and progression-free survival in patients with OC was not evaluated in our analysis; however, owing to the poor prognosis and short post-progression survival period of this disease, the progression-free survival was similar to overall survival (57, 58). In addition, according to previous similar research (8, 29, 59), we analyze all-cause deaths rather than OC-specific deaths in the present study, because research shows that there is little difference between them (60). Last, although we have comprehensively adjusted for potential confounding covariates in multivariate models, these findings still may be affected by unknown or unmeasured confounders including different clinical treatment and types of mutation (61, 62). Additionally, residual confounding from unmeasured confounders as well as the assessment of dietary intake may also influence the results, since these variables were assessed base on self-administered questionnaires.

## Conclusion

CVs are commonly consumed worldwide, and they are rich in nutrients, such as ITCs and antioxidants. In the present study, increasing pre-diagnosis consumption of CVs was strongly associated with better survival in patients with OC. This protective effect was similar in the analysis of ITCs from CVs. Nevertheless, further studies with longer follow-up periods are needed to confirm our findings.

## Data Availability Statement

The original contributions presented in the study are included in the article/Supplementary Material, further inquiries can be directed to the corresponding author/s.

## Ethics Statement

The studies involving human participants were reviewed and approved by the Institutional Review Board of the Ethics Committee of Shengjing Hospital of China Medical University, Shenyang, China. The patients/participants provided their written informed consent to participate in this study.

## Author Contributions

Q-JW, Y-HZ, and T-TG: contributed to the study design. SG, X-QL, SY, and T-TG: collection of data. Y-FW and F-HL: analysis of data. Y-FW, Y-YH, F-HL, Z-YW, H-YW, SZ, ML, and Q-JW: wrote the first draft of the manuscript and edited the manuscript. All authors have read and approved the final manuscript.

## Funding

This work was supported by the National Key R&D Program of China (No. 2017YFC0907404 to Y-HZ), the Natural Science Foundation of China (No. 82073647, 81602918 to Q-JW, and 82103914 to T-TG), China Postdoctoral Science Foundation Funded Project (No. 2018M641752 to Q-JW), LiaoNing Revitalization Talents Program (No. XLYC1907102 to Q-JW), Shenyang high level innovative talents support program (No. RC190484 to Q-JW), and 345 Talent Project of Shengjing Hospital of China Medical University (Q-JW).

## Conflict of Interest

The authors declare that the research was conducted in the absence of any commercial or financial relationships that could be construed as a potential conflict of interest.

## Publisher's Note

All claims expressed in this article are solely those of the authors and do not necessarily represent those of their affiliated organizations, or those of the publisher, the editors and the reviewers. Any product that may be evaluated in this article, or claim that may be made by its manufacturer, is not guaranteed or endorsed by the publisher.

## Abbreviations

CVs: cruciferous vegetables
ITCs: isothiocyanates
OC: ovarian cancer
BMI: body mass index
HR: hazard ratio
CI: confidence interval
FIGO: International Federation of Gynecology and Obstetrics
OOPS: the ovarian cancer follow-up study
FFQ: food frequency questionnaire.
