# Supplementary material for: Pre-diagnosis Cruciferous Vegetables and Isothiocyanates Intake and Ovarian Cancer Survival: A Prospective Cohort Study
Source: Front Nutr. 2021 Nov 24;8:778031. doi: 10.3389/fnut.2021.778031 (PMC8654276; doi:10.3389/fnut.2021.778031)
Supplement: Supplementary file 1 [file Data_Sheet_1.docx]

**Supplementary Table 1 Association between pre-diagnosis cruciferous vegetables intake and overall mortality among women diagnosed with ovarian cancer (n= 703)**

| **Cruciferous vegetables**  **(servings per week)** | **Deaths, N (% of total deaths)** | **Multivariate**  **HR (95% CI)** | **Multivariate**  **P for trend** |
| --- | --- | --- | --- |
| **Chinese cabbage** |  |  |  |
| None or < 1 | 35 (26.92) | 1.00 (Ref) | < 0.05 |
| 1- <4 | 73 (56.15) | 0.61 (0.39-0.95) |  |
| ≥ 4 | 22 (16.93) | 0.44 (0.23-0.81) |  |
| **Pakchoi** |  |  |  |
| None or < 1 | 43 (33.08) | 1.00 (Ref) | 0.07 |
| 1- <4 | 75 (57.69) | 0.97 (0.64-1.48) |  |
| ≥ 4 | 12 (9.23) | 0.46 (0.22-0.98) |  |
| **Kohlrabi** |  |  |  |
| None or < 1 | 74 (56.93) | 1.00 (Ref) | < 0.05 |
| 1- <4 | 49 (37.69) | 0.78 (0.52-1.16) |  |
| ≥ 4 | 7 (5.38) | 0.37 (0.15-0.88) |  |
| **Rape** |  |  |  |
| None or < 1 | 73 (56.15) | 1.00 (Ref) | 0.09 |
| 1- <4 | 52 (40.00) | 0.85 (0.57-1.28) |  |
| ≥ 4 | 5 (3.85) | 0.40 (0.14-1.14) |  |
| **Broccoli** |  |  |  |
| None or < 1 | 78 (60.00) | 1.00 (Ref) | 0.43 |
| 1- <4 | 42 (32.31) | 0.88 (0.59-1.31) |  |
| ≥ 4 | 10 (7.69) | 0.79 (0.39-1.62) |  |
| **Cauliflower** |  |  |  |
| None or < 1 | 85 (65.39) | 1.00 (Ref) | 0.15 |
| 1- <4 | 38 (29.23) | 0.99 (0.65-1.51) |  |
| ≥ 4 | 7 (5.38) | 0.56 (0.25-1.25) |  |
| **Raphanus sativus** |  |  |  |
| None or < 1 | 75 (57.69) | 1.00 (Ref) | 0.92 |
| 1- <4 | 48 (36.93) | 1.05 (0.70-1.58) |  |
| ≥ 4 | 7 (5.38) | 0.84 (0.35-2.01) |  |

CI, confidence interval; HR, hazard ratio; ref, reference.

Multivariate model adjusted for age at diagnosis, body mass index, comorbidities, diet change, education, FIGO stage, histological type, histopathologic grade, menopausal status, parity, physical activity, residual lesions, smoke status, meat, fruit, green leafy vegetables, allium vegetables, and total energy.

Multivariate P for trend for linear trend calculated from category median values.


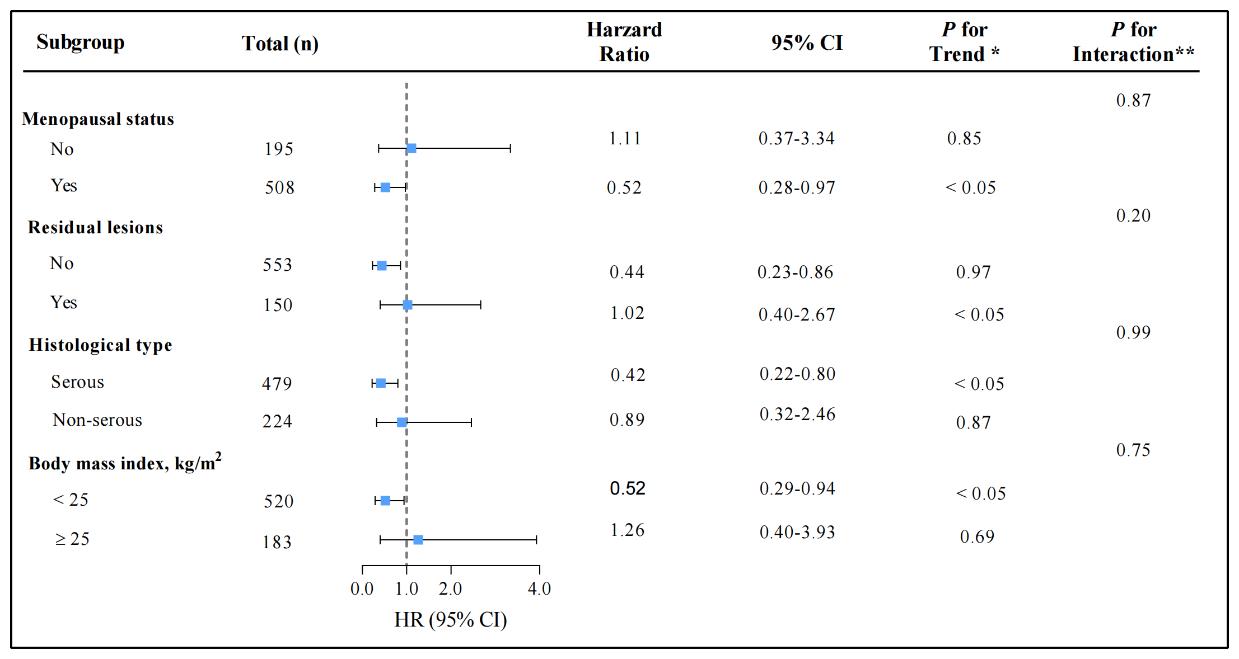


**Supplementary Figure 1 Overall survival among ovarian cancer patients for isothiocyanate intake across strata of various factors**

The analyses used three categories of isothiocyanate intake (T_1_＜3.90, T_2_ 3.90-9.44 and T_3_ ≥9.44µmol/day). The forest plot represents the HRs of the comparison of the highest versus the lowest of isothiocyanate intake. Cox model stratified by menopausal status, residual lesions, histological type and body mass index, with additional adjustments for age at diagnosis, body mass index, comorbidities, diet change, education, FIGO stage, histological type, histopathologic grade, menopausal status, parity, physical activity, residual lesions, smoke status, meat, fruit, green leafy vegetables, allium vegetables and total energy.

* indicates P for trend across levels of isothiocyanate intake.

** indicates P for interaction between strata and isothiocyanate intake.

P values are two-sided.
